# Ego-relevance in team production

**DOI:** 10.1371/journal.pone.0279391

**Published:** 2022-12-21

**Authors:** César Mantilla, Zahra Murad

**Affiliations:** 1 Economics Department, Universidad del Rosario, Bogotá, Colombia; 2 Accounting, Economics and Finance, University of Portsmouth, Portsmouth, United Kingdom; 3 UNEC Cognitive Economics Center, Azerbaijan State University of Economics, Baku, Azerbaijan; Sapienza University of Rome, ITALY

## Abstract

We study how individuals’ effort contribution to a team production task varies depending on whether the task is ego relevant or not. We conduct an experiment to test the effect of ego-relevance when the team production depends on the team’s top- or bottom-performer. Ego-relevance is manipulated by calling the Raven IQ Test an “IQ Task” or a “Pattern Task.” We find that the effort contributed to the task is affected by ego-relevance and the impact of the team production function on effort contribution is mediated by the teammate’s expected effort contribution. Ego-relevance increases the responsiveness to the teammate’s expected effort contributions. Similarly, more responsive behavior is noticeable when the team production depends on the bottom-performer. However, we do not observe interaction-effects between ego-relevance and the team production function that affect effort contributions.

## Introduction

Team production environments are generally prone to free-riding and shirking among teammates [1, 2]. However, the literature is silent on how this may depend on the ego-relevance of tasks in a team project. A familiar example is preparing a co-authored academic project. One could easily distinguish between tasks that would affect one’s ego depending on success or failure (e.g., writing a mathematical proof or programming a statistical test) and tasks whose completion would not affect ego (e.g., producing a project’s expense report).

The standard economic theory makes no distinctions concerning the ego-relevance of the team production task that individuals are required to undertake. However, behavioral economics and psychology predict that ego-relevance may boost or hinder individual contributions to a team task through separate mechanisms. On the one hand, ego-relevance may stimulate the willingness to contribute costly effort by raising individuals’ self-confidence and intrinsic motivation [3–5]. On the other hand, ego-relevance may demote this willingness to contribute effort because of ego-protection. This behavior, known in the literature as a self-handicapping motive in effort and task selection [6, 7], predicts that fear of failing the task might reduce individuals’ effort to justify their non-fulfillment instead of receiving a signal of low ability or incompetence. The relationship between ego-relevance, confidence levels, and effort choices has been studied before in individual decision-making settings [8, 9], whereas the tests of ego-relevance effects in strategic settings are scarce [10]. Our paper provides one of the first direct tests of ego-relevance effects on team production.

Higher confidence about one’s abilities is related to more self-interested status-seeking behavior [11]. Nonetheless, it is unclear whether the effect of ego-motivation is robust to the strategic interactions within a team production setting. Ego-motivation might boost contribution effort by allowing team members to feel that they “pull the team to get the job done.” The other side of ego-relevance, namely ego-protection, may hinder effort by providing a self-justification to shirk. Ego-motivation and ego-protection may coexist for a given production function, and the activation of one or the other may depend on the team members’ perceived ability to perform a task. However, whether we consider teammates’ contribution efforts as complements or substitutes could make one side of ego-relevance more salient than the other. Complementary efforts may foster ego-motivation gains from “pulling one’s team” by increasing contributions. Substitutable efforts may trigger ego-protection in settings where a justification for effort reduction is not wanting to learn that one was incompetent [12, 13].

We design an experiment to test the effects of ego-relevance of the task on contribution decisions and its association with the team production function. Our experiment employs a 2x2 factorial design. In one dimension, we manipulate the ego-relevance of the task by framing the 10-item Progressive Raven Matrix task either as an “IQ task” (*ego*) or “Pattern task” (*non-ego*). In the other dimension, we manipulate the team production function: it depends on the team’s maximum individual contribution, which we call *best-shot* production; or it depends on the team’s minimum individual contribution, which we call *complementary* production. Whereas the latter evokes a coordination equilibrium, the former evokes an anti-coordination equilibrium.

Given the complexity of combining ego-relevance with strategic concerns, we opt for a one-shot strategic interaction and rely on measuring incentivized beliefs about teammate’s expected contributions. The role of beliefs as essential predictors of effort contribution decisions in social dilemmas is not new: they are significant predictors of transfers in public good games [14–17] and trust games [18]. In a game with multiple equilibria, players’ beliefs about other players’ strategies are important for equilibrium selection [19–22]. In exchange for simplifying strategic concerns, this design feature limits our study to scenarios without reciprocation of past actions and without opportunities to infer how ego-relevant the task is for a teammate.

We find that the effects of ego-relevance and the team production function on the contribution decisions are mediated by conditional responses to the teammate’s expected contribution. We show that ego-relevance magnifies the effect of expectations regarding the teammate’s contribution: among the most pessimistic participants (i.e., those expecting a low contribution from their teammate), contributions are lower in the *ego* than in the *non-ego* treatment. The effect of ego-relevance is reverted among the optimistic participants (i.e., those expecting a high contribution from their teammate), with contributions being higher in the *ego* than in the *non-ego* treatment.

Our results speak to two strands of the literature. First, to the study of how ego-relevance affects economic decisions. This literature has focused on how the ego-relevance induced by a task or decision-making context affects belief updating and information processing. For instance, wishful thinking might be simultaneously driven by ego-related motives and non-ego-related motives such as optimism [23]. The conclusions from studies involving feedback are somewhat mixed. People often ignore positive [24, 25] and negative information [26, 27]. There is also evidence of asymmetry when incorporating positive or negative information into updating their ego-relevant traits [28–30]. Differently from all of these studies, we test whether the ego-relevance of a task affects individual decisions in a *strategic* setup. Our findings point out a connection between ego-relevance and the conditional contribution of effort in a team task. This evidence is important in the light of theoretical models connecting principal-agent relationships with self-esteem and the reaction to ego-threats [31]. Moreover, it has implications for real-world decision makers, such as managers in organizations, in motivating effort in team production tasks of varying ego-relevance, which we discuss later in our paper.

Second, we contribute to the literature exploring how team production functions affect effort and how it interacts with the ego-relevance of the task. Although sports data provide quasi-experiments for testing how individuals contribute to their teams [32–34], specific mechanisms can be more easily tested using controlled experiments. For instance, there has been considerable laboratory work testing how sorting heterogeneous agents into teams affects effort contributions [2, 35, 36]. A study with similar team production incentives to ours, although embedded in a between-teams contest, shows that effort contributions are very different (even if group composition is similar) when the team production is modeled as best-shot or as a weak-link (i.e., complementary efforts) function [37]. Whereas the former production model induces free-riding among the weak players within the group, all participants contribute similar effort levels in the latter. In our study, we find much smaller differences in participants’ behavior between these two production functions, probably due to our setting’s one-shot nature. By studying the interaction of ego-relevance with the type of team production function, we present a fuller picture of whether the effect of ego-relevance on effort contributions depends on the team production function.

## Model

A team consisting of two members, *i* and *j*, must undertake a team task whose payoff is negatively affected by the individual effort allocation and positively affected by a team score. Team members contribute to the team score by correctly solving a number of subtasks *τ* ∈ {0, 1, 2, …, *T*}. The effort contribution decision consists on deciding how many of the *T* subtasks will be “activated” (i.e., accounted in the score) at a cost of *c* per subtask to be subtracted from an endowment *e*. We define the activation decision as *A*_*k*_ ∈ {0, 1, 2, …, *T*}, ∀*k* ∈ {*i*, *j*}.

To give room to ego-motivation and ego-protection to operate, we differentiate the *intended* effort contribution, *A*_*k*_, from the *effective* effort contribution, which also depends on the ability in solving the subtasks. The key element of the effective contribution is that it is not known by the participants when making their effort choice (and, in our setting, it was only revealed at the end of the experiment in exchange for a payment). We thus define this effective contribution as the individual score *S*_*k*_ = *ω*_*k*_*A*_*k*_, ∀*k* ∈ {*i*, *j*}. Here, *ω*_*k*_ ∈ [0, 1] is the participant’s *k* ability to solve a subtask *τ* (with subtasks assumed to be identical for simplicity).

Eq 1 depicts the payoffs of the team task as a function of individual effort costs, where *c* can be interpreted as the unit cost of effort; and the team’s score *S*, where each additional point yields a benefit to the team of *b* units.

$$\begin{array}{c} \pi_{k}=e-cA_{k}+bS\;\;\;\;\;\;\;\;\;\;\forall k\in \left\{i,j\right\} \end{array}$$ (1)

Finally, we explain how the individual scores, *S*_*i*_ and *S*_*j*_, are aggregated into the team score *S*. In the experiment, we implement two different production technologies, mimicking different types of team production tasks. First, we have *complementary* production, where *S* = min(*S*_*i*_, *S*_*j*_). This technology represents joint tasks depending on the contribution of the weakest link. It is also called the “O-ring” production, referring to the importance that every piece–or member–has in the final team production level, in an analogy to the malfunction of O-rings that lead to the failure of the Challenger shuttle [38]. Second, we have the *best-shot* production, where *S* = max(*S*_*i*_, *S*_*j*_). This technology is useful for representing joint tasks where team production is better represented by the largest individual contribution, aligned with the effect of “superstars” [39]. This might be the case of creative industries, where one of the teammates’ ideas is implemented by the entire team. We will now explore the predictions for each production technology.

### Complementary production

In our experiment, we match participants according to their ability in homogeneous groups. We thus assume an identical ability parameter within each group, or *ω* = *ω*_*i*_ = *ω*_*j*_. Let us also define *ωb* as the net benefit, with *ω* ∈ [0, 1] imposing an “imperfect ability penalty” on the expected benefit when *ω* falls below one. As long as the net benefit exceeds the activation cost (i.e., *ωb* > *c*), the participant *i*’s activation decision is not trivially defined by her low ability, which would have led her to set *A*_*i*_ = 0 regardless of her teammate’s expected activation. With this in mind, the payoff function has the form:

$$\begin{array}{c} \pi_{k}=e-cA_{k}+\omega b\cdot \text{min}\left(A_{i},A_{j}\right)\;\;\;\;\;\;\;\;\;\;\forall k\in \left\{i,j\right\} \end{array}$$

This incentive structure resembles a minimum-effort game where every symmetric contribution decision is an equilibrium in a coordination game [40]. To see why, let us depart from the symmetric activation profile where player *i* mimics *j*’s activation level, or (*A*_*j*_, *A*_*j*_). Any upward deviation (i.e., *A*_*i*_ = *A*_*j*_+ *δ*) will be unprofitable because the team score is still determined by *ωA*_*j*_ whereas *i* will pay an additional cost (i.e., *δc*). A downward deviation (i.e., *δ* < 0) will not be profitable either: the condition *ωb* > *c* guarantees that participant *i* is better off activating subtasks as long as they positively affect the team score. In the calibration shown below, the benefit *b* is 2.5 times the cost *c*. Hence, the condition *ωb* > *c* will hold for ability levels above *ω* = 0.4. We show in Table 3 that the average ability in our sample is twice this value, giving us some confidence that participants are very likely to meet this condition. Conversely, any asymmetric profile is not an equilibrium because the participant with the larger activation level will deviate downward.

**Table 3:** Descriptive statistics by treatment.

	Ego-relevant				Non Ego-relevant			
	Complementary		Best-shot		Complementary		Best-shot	
**Part 1: Initial Raven Matrices Test**								
Correct matrices (out of 10)	8.24	(1.50)	8.00	(1.72)	8.20	(1.51)	7.88	(1.59)
Belief about own score	7.80	(1.57)	7.55	(1.80)	7.93	(1.49)	7.80	(1.52)
Confidence top half	71.08	(21.05)	70.63	(22.89)	72.84	(21.86)	70.53	(23.15)
Confidence top quarter	55.92	(28.54)	55.18	(27.83)	57.88	(28.33)	56.94	(27.33)
**Part 2: Team Production**								
Allocation decision	6.39	(2.72)	6.13	(2.64)	6.49	(2.68)	6.40	(2.69)
Correct matrices (out of 10)	7.33	(1.83)	7.28	(1.87)	7.39	(1.84)	7.14	(1.51)
Belief about teammate’s allocation	6.47	(2.12)	6.16	(2.17)	6.27	(2.49)	6.13	(2.39)
**Earnings**								
Paid to learn score	35.8%		34.3%		18.9%		22.1%	
Payoff	1.19	(0.48)	1.84	(0.45)	1.25	(0.45)	1.81	(0.51)
**Demographics**								
Female	60.5%		59.7%		54.4%		62.1%	
Age	21.26	(2.22)	21.19	(2.22)	21.59	(2.27)	21.52	(2.26)
Taken Raven test before	14.9%		16.4%		9.5%		12.3%	
Oneness scale	2.66	(1.76)	2.39	(1.65)	2.78	(1.68)	2.51	(1.53)
Observations	148		140		148		154	

Reported values correspond to means, unless percentages indicate a proportion. Standard deviations in parentheses.

### Best-shot production

Given the assumption of identical ability within the group, the payoff function is:

$$\begin{array}{c} \pi_{k}=e-cA_{k}+\omega b\cdot \text{max}\left(A_{i},A_{j}\right)\;\;\;\;\;\;\;\;\;\;\forall k\in \left\{i,j\right\} \end{array}$$

With the team score being by the maximum (rather than the minimum) contribution, participants have incentives to anti-coordinate (rather than to coordinate). Hence, the activation profiles (*T*, 0) and (0, *T*) are equilibria in pure strategies. Note that when (*A*_*i*_, *A*_*j*_) = (*T*, 0), participant *i* will not deviate downward because *ωb* > *c* guarantees that profit maximization occurs with the maximum activation level. On the other hand, participant *j* will not deviate upward (i.e., *A*_*j*_ = *δ*) because *ωT* still determines the team score, and *j* will pay an unnecessary cost (i.e., *δc*).

Any other asymmetric profile cannot be an equilibrium in pure strategies. The player with the highest activation level will deviate upward to maximize her benefits, and the other will deviate downward to avoid wasting activation costs. Under the same reasoning, though with higher exposure to a coordination failure, deviations from symmetric activation profiles are also profitable.

### Ego-motivation and ego-protection

We require three assumptions to introduce the notions of ego-motivation and ego-protection into the model described above. First, we assume that ego-motivation emerges with high ability levels in a given task [3, 5], whereas ego-protection emerges with low ability levels in the same task [5–7]. Second, we assume that ego-motivation increases the benefit derived from the team score *S* [4], whereas ego-protection increases the costs from each contributed unit in *A*_*k*_. The reason is that each contributed effort unit will increase the probability of learning that one has failed. Third, we assume that individual contributions to the team score cannot be perfectly observed. This is plausible given the team production function with unobservable individual efforts, yielding more room for ego-motives to operate (i.e., participants cannot validate whether they are effectively contributing more or less than their teammate).

The augmented model, including these assumptions to include ego-relevance, goes as follows:

$$\begin{array}{c} \pi_{k}=\{\begin{array}{cc} e-cA_{k}+\omega \left(b+\mu \right)\cdot g\left(A_{i},A_{j}\right) & \text{if}\;\omega \geq \tilde{\omega }\\ e-\left(c+\varphi \right)A_{k}+\omega b\cdot g\left(A_{i},A_{j}\right) & \text{if}\;\omega <\tilde{\omega }, \end{array} \end{array}$$ (2)

where *μ* is the non-material benefit derived from ego-motivation when a participant *k*’s ability, *ω*, is above a threshold $\tilde{\omega }$; and *φ* is the non-material cost associated with ego-protection when $\omega <\tilde{\omega }$. This non-material cost can be interpreted as a psychological cost of anticipating failure. We implicitly assume that ego-motivation and ego-protection cannot operate simultaneously, as they depend on whether the participant’s ability falls above or below $\tilde{\omega }$, which we interpret as a “maximum difficulty” threshold. An ability *ω* below this threshold implies that the task is sufficiently hard to “reverse” the role of ego-relevance. It no longer motivates participants to perform the task but instead makes them avoid allocating additional effort to a task that will likely fail, causing feelings of incompetence.

In Eq 2, *g*(⋅) captures the team production function, either *best-shot* or *complementary*. Note that *μ* increases the marginal benefit of each additional unit scored by the team, and *φ* increases the marginal cost of each unit contributed in *A*_*k*_. Therefore, we can write $\tilde{b}=b+\mu$ as the augmented marginal benefit, accounting for the benefits of ego-motivation; and $\tilde{c}=c+\phi$ as the augmented marginal cost, accounting for the costs of ego-protection.

When explaining the production functions, the condition yielding an increase in *A*_*i*_ (up to *A*_*j*_ with the *complementary* function and to *T* with *best-shot*) was *ωb* > *c*. In the presence of ego-motivation, the reasoning is similar, though we employ $\tilde{b}$ and $\tilde{c}$ in the analysis instead of *b* and *c*. When ego-motivation is at play, the condition $\omega \tilde{b}>c$ becomes easier to meet. As an alternative interpretation, the minimum ability level required to choose *A*_*i*_ > 0 becomes lower. We predict the opposite behavior when ego-protection is at play. The minimum ability yielding *A*_*i*_ > 0 increases because $\omega b>\tilde{c}$ raises the required minimum ability.

In Eq 2 we implicitly assume that the benefits of ego-motivation, captured through *μ*, are symmetric within a team. This assumption explains why we put *μ* outside the production function. However, inferring asymmetries in the task’s ego-relevance may lead to strategic behavior or alternative predictions. For instance, the case where a participant *i* cares about ego-motivation and she assumes that *j* does not. The benefit from the team score will be *ωbg*(*μA*_*i*_, *A*_*j*_), with *μ* > 1. With the *best-shot* production function, the *max* argument causes that the ego-motivated player breaks the allocation symmetry upwards, as *μ* operates as a boost in perceived ability that raises the team score. By contrast, with *complementary* production, the boost in perceived ability may cause a reduction in the contribution from the ego-motivated player since a smaller contribution is compensated by *μ* > 1. Our one-shot setting minimizes the likelihood that these more complex behaviors arise by ruling-out teammates’ interactions.

## Experimental setup

In this Section, we present our experimental paradigm and explain how it is connected with the model. We then proceed to list our predictions and describe the data collection procedure.

### Experimental paradigm

**Fig 1:**
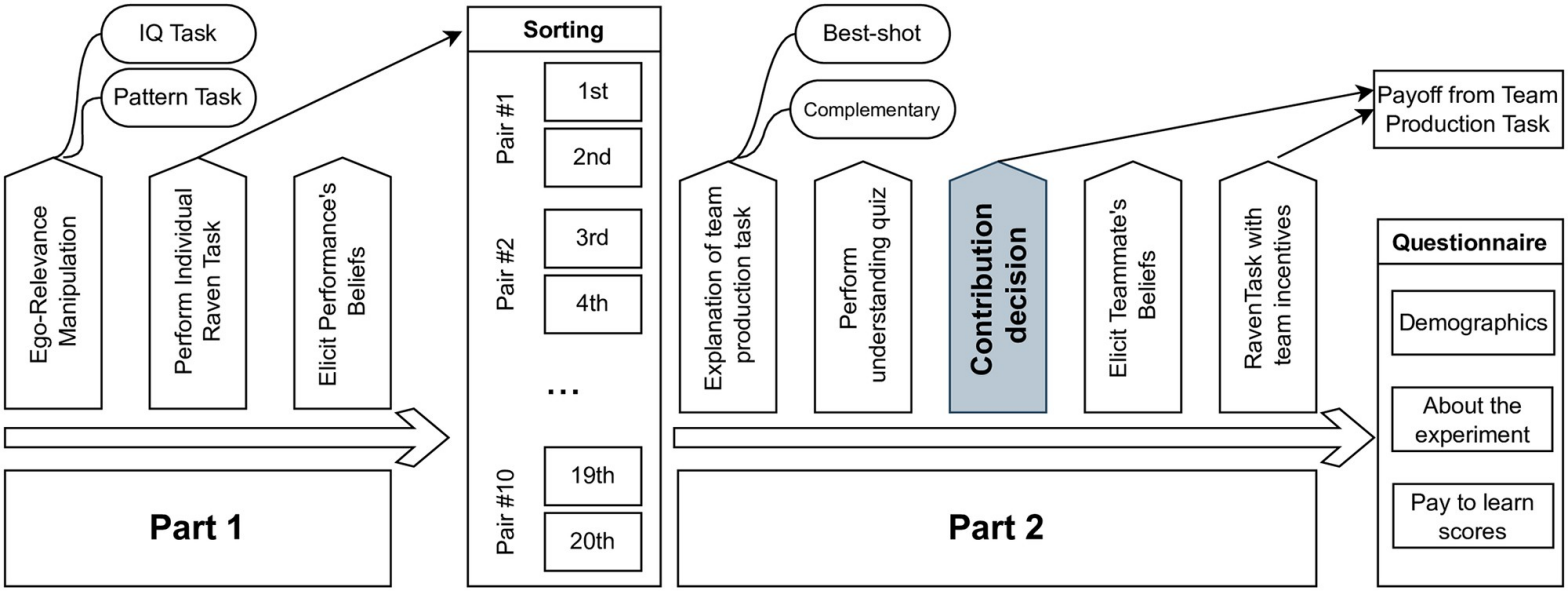
Timeline of steps within each part of the experiment.

Our experimental paradigm consists of two parts, described below, plus a final questionnaire. Fig 1 describes the timeline of the experiment. See the Online Supplementary Materials for the full instructions (also available at dx.doi.org/10.17504/protocols.io.cddms246).

#### Part 1

Participants were asked to complete a 10 Raven Progressive Matrices test within two minutes [41]. We announced that this test was not directly incentivized, but their performance would affect the quality of their teammate in Part 2 (i.e., they knew that the more matrices they correctly solved, the better their future teammate). We did not provide any other information about Part 2 during Part 1. Thus, we eliminated any possibility of hedging or intentional under-performance related to the teammate matching.

After completing Part 1, we elicited participants’ confidence in their performance. As a measure of *absolute* confidence, we asked for participants’ beliefs about their own score. We incentivized this belief by paying participants an additional £0.10 if their answer was correct. To measure *relative* confidence, we asked for the participant’s beliefs about their score being in the top half and top quarter of the distribution. Our purpose for measuring performance beliefs was twofold. First, to calibrate the parameter *ω* in our derivation of equilibria (see Tables 1 and 2). Second, we used these beliefs to control for overconfidence in our regression analysis, given the importance of this trait in the belief-updating process [23, 29], and in the comparison with teammates in terms of productivity [10].

**Table 1:** Payoff matrix for complementary production, with *ω*_*i*_ = *ω*_*j*_ = 0.8.

	*A*_*j*_ = 10	*A*_*j*_ = 9	*A*_*j*_ = 8	*A*_*j*_ = 7	*A*_*j*_ = 6	*A*_*j*_ = 5	*A*_*j*_ = 4	*A*_*j*_ = 3	*A*_*j*_ = 2	*A*_*j*_ = 1	*A*_*j*_ = 0
*A*_*i*_ = 10	**2;2**	1.8;1.9	1.6;1.8	1.4;1.7	1.2;1.6	1;1.5	0.8;1.4	0.6;1.3	0.4;1.2	0.2;1.1	0;1
*A*_*i*_ = 9	1.9;1.8	**1.9;1.9**	1.7;1.8	1.5;1.7	1.3;1.6	1.1;1.5	0.9;1.4	0.7;1.3	0.5;1.2	0.3;1.1	0.1;1
*A*_*i*_ = 8	1.8;1.6	1.8;1.7	**1.8;1.8**	1.6;1.7	1.4;1.6	1.2;1.5	1;1.4	0.8;1.3	0.6;1.2	0.4;1.1	0.2;1
*A*_*i*_ = 7	1.7;1.4	1.7;1.5	1.7;1.6	**1.7;1.7**	1.5;1.6	1.3;1.5	1.1;1.4	0.9;1.3	0.7;1.2	0.5;1.1	0.3;1
*A*_*i*_ = 6	1.6;1.2	1.6;1.3	1.6;1.4	1.6;1.5	**1.6;1.6**	1.4;1.5	1.2;1.4	1;1.3	0.8;1.2	0.6;1.1	0.4;1
*A*_*i*_ = 5	1.5;1	1.5;1.1	1.5;1.2	1.5;1.3	1.5;1.4	**1.5;1.5**	1.3;1.4	1.1;1.3	0.9;1.2	0.7;1.1	0.5;1
*A*_*i*_ = 4	1.4;0.8	1.4;0.9	1.4;1	1.4;1.1	1.4;1.2	1.4;1.3	**1.4;1.4**	1.2;1.3	1;1.2	0.8;1.1	0.6;1
*A*_*i*_ = 3	1.3;0.6	1.3;0.7	1.3;0.8	1.3;0.9	1.3;1	1.3;1.1	1.3;1.2	**1.3;1.3**	1.1;1.2	0.9;1.1	0.7;1
*A*_*i*_ = 2	1.2;0.4	1.2;0.5	1.2;0.6	1.2;0.7	1.2;0.8	1.2;0.9	1.2;1	1.2;1.1	**1.2;1.2**	1;1.1	0.8;1
*A*_*i*_ = 1	1.1;0.2	1.1;0.3	1.1;0.4	1.1;0.5	1.1;0.6	1.1;0.7	1.1;0.8	1.1;0.9	1.1;1	**1.1;1.1**	0.9;1
*A*_*i*_ = 0	1;0	1;0.1	1;0.2	1;0.3	1;0.4	1;0.5	1;0.6	1;0.7	1;0.8	1;0.9	**1;1**

**Table 2:** Payoff matrix for best-shot production, with *ω*_*i*_ = *ω*_*j*_ = 0.8.

	*A*_*j*_ = 10	*A*_*j*_ = 9	*A*_*j*_ = 8	*A*_*j*_ = 7	*A*_*j*_ = 6	*A*_*j*_ = 5	*A*_*j*_ = 4	*A*_*j*_ = 3	*A*_*j*_ = 2	*A*_*j*_ = 1	*A*_*j*_ = 0
*A*_*i*_ = 10	2;2	2;2.1	2;2.2	2;2.3	2;2.4	2;2.5	2;2.6	2;2.7	2;2.8	2;2.9	**2;3**
*A*_*i*_ = 9	2.1;2	1.9;1.9	1.9;2	1.9;2.1	1.9;2.2	1.9;2.3	1.9;2.4	1.9;2.5	1.9;2.6	1.9;2.7	1.9;2.8
*A*_*i*_ = 8	2.2;2	2;1.9	1.8;1.8	1.8;1.9	1.8;2	1.8;2.1	1.8;2.2	1.8;2.3	1.8;2.4	1.8;2.5	1.8;2.6
*A*_*i*_ = 7	2.3;2	2.1;1.9	1.9;1.8	1.7;1.7	1.7;1.8	1.7;1.9	1.7;2	1.7;2.1	1.7;2.2	1.7;2.3	1.7;2.4
*A*_*i*_ = 6	2.4;2	2.2;1.9	2;1.8	1.8;1.7	1.6;1.6	1.6;1.7	1.6;1.8	1.6;1.9	1.6;2	1.6;2.1	1.6;2.2
*A*_*i*_ = 5	2.5;2	2.3;1.9	2.1;1.8	1.9;1.7	1.7;1.6	1.5;1.5	1.5;1.6	1.5;1.7	1.5;1.8	1.5;1.9	1.5;2
*A*_*i*_ = 4	2.6;2	2.4;1.9	2.2;1.8	2;1.7	1.8;1.6	1.6;1.5	1.4;1.4	1.4;1.5	1.4;1.6	1.4;1.7	1.4;1.8
*A*_*i*_ = 3	2.7;2	2.5;1.9	2.3;1.8	2.1;1.7	1.9;1.6	1.7;1.5	1.5;1.4	1.3;1.3	1.3;1.4	1.3;1.5	1.3;1.6
*A*_*i*_ = 2	2.8;2	2.6;1.9	2.4;1.8	2.2;1.7	2;1.6	1.8;1.5	1.6;1.4	1.4;1.3	1.2;1.2	1.2;1.3	1.2;1.4
*A*_*i*_ = 1	2.9;2	2.7;1.9	2.5;1.8	2.3;1.7	2.1;1.6	1.9;1.5	1.7;1.4	1.5;1.3	1.3;1.2	1.1;1.1	1.1;1.2
*A*_*i*_ = 0	**3;2**	2.8;1.9	2.6;1.8	2.4;1.7	2.2;1.6	2;1.5	1.8;1.4	1.6;1.3	1.4;1.2	1.2;1.1	1;1

We did not incentivize the relative confidence measures because the instrument was already complex and lengthy for online experiment standards. There is evidence that the lack of incentives or very short information on incentive structures improves the additivity and accuracy of beliefs compared to more complex scoring rule instructions [42, 43]. We expected that lengthy instructions for additional scoring rules would have increased dropouts and hence decided against using incentives.

We did not restrict *relative* confidence levels to be higher in the top half than in the top quarter and 79 participants (13.4%) violated this condition. A robustness check for the main regression, excluding these participants, yields results qualitatively similar while preserving the statistical significance of the variables of interest.

#### Part 2

In Part 2, participants were asked to complete another 10 Raven Progressive Matrices test within two minutes, but now they were grouped in pairs. The matching procedure went as follows. Participants were ranked from first to twentieth based on their performance in Part 1, and then they were matched consecutively. That is, the first and the second were paired, the third and the fourth were paired, and so on (ties were randomly broken). We informed participants about the matching procedure, but they did not receive any information regarding their position in the ranking. The matching procedure ensured that we had homogenous teams in terms of ability, and the uncertainty about teammate’s ability was minimal.

We opted for this homogeneous matching because, as mentioned in the previous section, it simplifies our predictions in an already complex framework by letting us assume that the ability within the group is the same. Participants can be assured that their teammate has the closest possible ability level to their own, simplifying their decision environment. However, this design choice comes at a cost: we are blocking any mechanism in which ego-relevance depends on the participant’s beliefs about considerable differential abilities within the team. We thus aim to observe the effects of ego-relevance when responding to the beliefs about the efforts of a highly similar teammate. In the last section of our paper, we return to this discussion and its implications regarding external validity.

Pairs were then randomly assigned to a team production technology, either the *best-shot* or the *complementary* production function. The payoff function follows Eq 1, with the parameters *e* = £1, *c* = £0.1, and *b* = £0.25. The effort contribution decision was explained as the number of “activated” questions, from zero to ten, or *A*_*k*_ ∈ {0, 1, …, 9, 10}. The activation decision can be thought of as an *intended* contribution since it defined how many matrices, but not which ones (and specially not the correctly solved), would be randomly selected as part of the scoring subset from the participant *i*.

By contrast, the *effective* effort contribution from participant *i* would be the number of correctly solved matrices within the scoring subset (*S*_*i*_). In the computation of *S*_*k*_, each correctly solved matrix within the scoring subset counted as one additional point. If the time ran out, the unsolved matrices were also eligible to be part of the scoring subset, meaning their contribution to the score was null. This “noise” in the effective effort contribution lets the ego-protection (resp. ego-motivation) mechanism operate: participants did not need to validate whether their contribution translated into a lower (resp. higher) team production, so they could self-deceive into thinking that they did not waste individual resources in a difficult task (resp. that they were pulling their team’s production upward).

Given that participants could not select which matrices would enter the scoring subset, they could not hedge between their contribution decision and their performance in the task: as long as a participants activated at least one matrix, they had an incentive to correctly complete as many matrices as possible. This design feature ensured that participants had a financially costly effort contribution decision, yet the participants’ performance in the task acted as a “real-effort” component. There is some evidence that contribution to a public good is similar whether the effort is induced, trivial, or useful [44]. Considering the ego-related nature of our research question, and following recent advocacy for real-effort tasks [45], we adopt induced effort combined with real effort to measure effort decisions in our experiment.

We opted for having only one contribution decision in Part 2 for two reasons. First, the effects of ego-motivation may be harder to identify in a dynamic setting. Participants may become more strategic given our fixed matching based on similar abilities and, simultaneously, the repetition of the tasks may decrease the effects of ego-motivation (e.g., due to belief-updating regarding confidence in their ability, or because repetition makes the task more tedious and therefore less ego-relevant). Second, since the study was conducted on Prolific, we designed a relatively short instrument to avoid high dropout rates. An additional round would have taken at least three minutes (for solving the Raven matrices and making the decision), a non-negligible amount of time in this platform.

Participants knew they would not receive information about their individual scores or their teammate’s contribution, but only about their payment. This ensured sufficient uncertainty to allow the ego-motivation and the ego-protection mechanisms to operate: participants can self-deceive regarding the random selection of matrices graded to compute the score; and whether it is their own or their teammate’s score that determines the team score. Participants had to correctly respond to a control quiz checking their understanding of the instructions and the payoff mechanism in an open-ended format. If they did not answer correctly, they could not proceed with the contribution decisions and would be paid a participation fee without a bonus.

We elicited beliefs regarding their teammate’s contribution using a simple incentivized procedure (receiving a bonus of £0.20 if their guess was correct). This was done on the same page where participants submitted their effort contribution decision. They then proceeded with the second Raven test that would determine the team outcome and their payoffs.

#### Final questionnaire

After the Raven matrices from Part 2 were completed, participants had to fill out a questionnaire on demographics, their previous knowledge about Raven Matrices, how close they felt to their teammate (by eliciting the Oneness Scale employed in [46]), and open-ended questions regarding their thoughts about the experiment. As a manipulation check for ego-relevance, we asked whether they would like to pay to be informed about their task scores from Part 1 and Part 2. Each piece of information would cost £0.10 to be subtracted from their final payments. If our framing of the task affected ego-relevance, we would observe different rates of those paying to learn their scores.

### Treatment arms

Our experiment employed a 2x2 between-subjects design. We manipulated the type of team production function and the ego-relevance of the task. As we already explained the former, let us focus on the latter.

We employed a novel ego-relevance manipulation where we kept the task constant, but we changed the framing of the task description. In the *non-ego* treatment, the instructions told participants that they would be shown ten patterns with a missing element, and their task was to select the option that completes the pattern. In the *ego* treatment, we raised the ego-relevance of the task by additionally telling participants that the task was taken from an Intelligence Quotient (IQ) test and referred them to a published paper that showed a significant relationship between IQ and life outcomes [47]. Similarly, a recent study achieved low-ego manipulation by presenting participants with a published paper showing no relationship between IQ and life outcomes [30]. Throughout the experiment, we referred to the task as the “Pattern Task” in the *Non-Ego* treatment and the “IQ task” in the *ego* treatment. Both labels for the task were accurate and did not involve deception or misleading information.

### Equilibrium predictions and hypotheses

Tables 1 and 2 display the payoff matrices under the *complementary* and *best-shot* production, respectively. For this calibration, we set an average ability of *ω* = 0.8. This value is similar to the elicited absolute confidence beliefs in Part 1 of our experiment (i.e., before the joint production task), as reported in Table 3.

In the treatment with *complementary* production, every symmetric contribution *A*_*i*_ = *A*_*j*_ is an equilibrium (see the bold cells in Table 1). The equilibria structure of minimum-effort games [40] is preserved as long as *ωb* > *c*. By rewriting this condition as *ω* > *c*/*b*, we introduce an ability threshold based on an “activation cost-benefit ratio” that defines whether participants should activate subtasks or not. For our calibration, we have *ω* ≥ 0.4. Any ability level *ω* ≤ 0.4 yields {*A*_*i*_, *A*_*j*_} = {0, 0} as the unique equilibrium.

In the *best-shot* treatment, the predicted equilibria correspond to the anti-coordination outcomes where {*A*_*i*_, *A*_*j*_} are equal to {0, 10} and {10, 0} if *ω* > 0.4 (see the bold cells in Table 2). One teammate activates all her subtasks (i.e., the ten Raven questions), and her teammate’s best response is a null activation. If *ω* = 0.4, any outcome {*A*_*i*_, 0} or {0, *A*_*j*_} is an equilibria. Finally, if the ability falls below the threshold (i.e., *ω* < 0.4), {0, 0} is the unique equilibrium.

The role of ego-relevance in the equilibrium predictions appears through a modification of the activation cost-benefit ratio in the expression *ω* > *c*/*b*. In the case of ego-motivation, the value for *ω* will be $c/\tilde{b}$. Since this value is lower than *c*/*b*, the null contribution equilibrium (i.e., {0, 0}) is less likely to occur. Imagine, for instance, that the ego-motivation utility is one-fifth of the monetary benefit *b*. The threshold defining the emergence of the {0, 0} equilibrium would drop from four-tenths to one-third. Ego-protection would have the opposite effect, as $\tilde{c}/b$ is larger than *c*/*b*. Imagine that the cost associated with ego-protection is one-fifth of *c*, and thus $\tilde{c}/b=0.48$. In this case, the {0, 0} equilibrium becomes more likely because the expected ability must exceed a higher threshold. With this intuition in mind, we derive our first prediction:

**Hypothesis 1 (H1):** *The ego-relevance leads to a higher frequency of more extreme (i.e., full and null) contributions in Part 2.*

Regarding the team production functions, we would also expect more extreme contributions *A*_*k*_ under the *best-shot* production than under the *complementary* production due to the multiplicity of equilibria in the latter case. Therefore, we derive additional testable hypotheses based on the players’ response to their beliefs about their teammate’s contribution, *A*_*k*_:

**Hypothesis 2 (H2):** *In the Complementary production treatment, participants’ contribution increases as their beliefs of their teammate’s contribution increases.*

**Hypothesis 3 (H3):** *In the Best-Shot production treatment, participants’ contribution decreases as their beliefs of their teammate’s contribution increases.*

Finally, the interaction effect of ego-relevance and the team production function is more convoluted. Note that Hypotheses H2 and H3 are written in terms of best-responses to the teammate’s expected contribution, and these best-responses go in opposite directions for the *best-shot* and the *complementary* production functions. We argue that, to the extent that ego-relevance alters the distance between ability and the cost-benefit threshold, the interaction effect needs to involve any possible difference in perceived ability with respect to the teammate. Hence, ego-relevance increases the responsiveness to the teammate’s expected contribution.

**Hypothesis 4 (H4):** *Ego-relevance amplifies the response to the teammate’s contribution beliefs. Under best-shot production, an increase in the teammate’s expected contribution decreases the participant’s contribution. Under Complementary production, an increase in the teammate’s expected contribution increases the participant’s contribution*.

### Data collection procedure

We obtained ethical approval from the University of Portsmouth, Faculty of Business and Law Ethics Committee (reference number BAL/2018/E518/MURAD). We recruited participants in April 2019 using the online platform www.prolific.co with a fixed payment of £2.00 per participant, plus a bonus payment determined by participants’ decisions. The bonus was, on average, £1.52 (± 0.56 std. dev.). Since the experiment lasted 15 minutes on average, the payment was equivalent to a £14 hourly rate. The initial screen of the experiment included a written consent form that participants needed to agree to proceed (see Section C in S1 Appendix). We pre-selected participants to be students within the age range of 18–25 and residing in the United Kingdom. The purpose was to have a homogeneous participant pool and a relatively homogeneous performance in the task. This sample targeting was common knowledge, as it was posted in the www.prolific.co announcement during recruitment. The experiment was conducted in oTree [48].

We calculated the sample size given an error probability of 5%, power of 80%, contribution ratio of 1-to-1, and predicted effect sizes of Cohen’s d = 0.25. The resulting required sample size was 416. In a pilot with 100 participants, we observed that the effect size was smaller than predicted. We proceeded to recalculate the required sample size using a linear multiple regression model R2 deviation from zero with small effect sizes (f2 = 0.02), the error probability of 5%, power of 80%, and three main predictors (ego-relevance, the team production function, and the interaction between these variables). The required sample size was 550. We collected 590 observations to account for possible outliers, failures in the comprehension quiz, and incomplete observations. Our random assignment to the four treatments yielded 140 participants in the *best-shot ego*, 154 in the *best-shot non-ego*, 148 in the *complementary ego*, and 148 in the *complementary non-ego* treatments.

## Results

In this section, we first present the descriptive results and proceed with the regression analyses. We report here all the treatment variations and experimental sessions we have conducted for this research question. The DOI number 10.17632/7mfdv9dr2x.1 stores the data for the replication of results.

### Descriptive statistics

Table 3 presents the descriptive statistics of our key variables of interest in Parts 1 and 2, and demographic characteristics by treatment condition. The non-parametric Mann-Whitney tests reveal that, before controlling for other covariates, there is no difference between the treatments in participants’ contribution decisions (*p* > 0.050). The distributions of contributions are displayed, by treatment, in Fig B.1 in S1 Appendix.

We do not find treatment differences in the performance in Parts 1 and 2. By contrast, we find that participants were more likely to pay for learning their scores in the *ego* treatment (35%) compared to the *non-ego* treatment (20%, Fisher exact test with *p* < 0.001). Section A in S1 Appendix provides a regression analysis validating that the *ego* treatment increases the probability of paying for the feedback of at least one of the tasks by 13 percentage points. This regression analysis also suggests that participants with lower confidence levels in their performance are more likely to pay.

Returning to Table 3, we do not report the individual or team scores because the production depends on the chosen subset of activated questions that will be accounted for when computing these scores. Nonetheless, the net payoff reveals that participants in the *best-shot* treatment earned, on average, £0.6 more than in the *complementary* production treatment (*t*-test with *p*−value <0.001). We do not find differences between treatments in the elicited demographic characteristics.

#### Does ego-relevance increase more extreme contributions?

**Fig 2:**
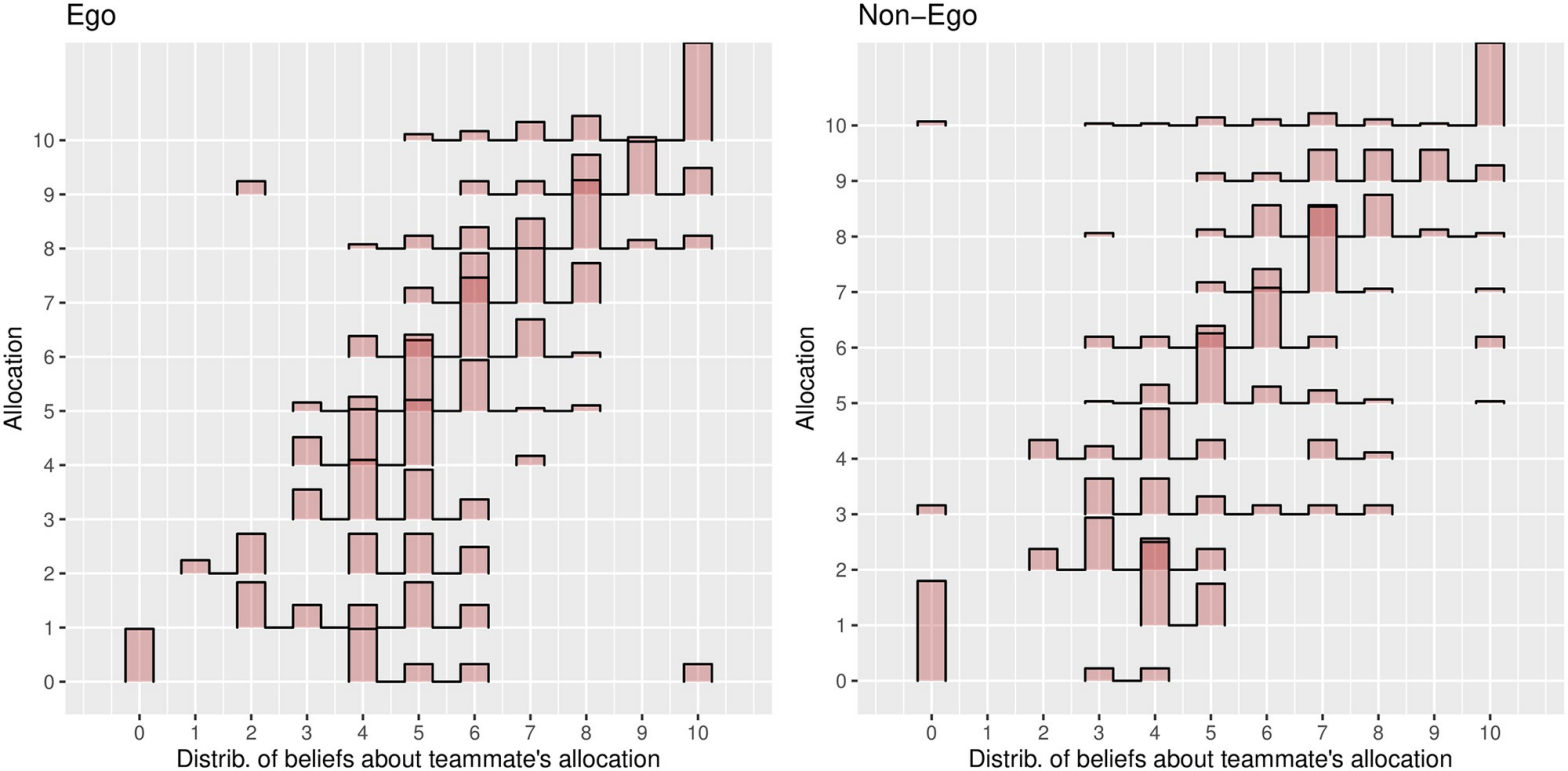
Effort contribution levels conditional on belief about teammate’s contribution. Panels correspond to ego-relevance treatment.

Our H1 postulates that ego-relevance will lead to a higher frequency of more extreme contributions (i.e., activation levels of 0 or 10). Fig 2 offers a visual inspection of this hypothesis, as it plots effort contribution decisions conditional on beliefs about the teammate’s contribution decision. Overall, we observe a low proportion of null contributions: 3.3% and 3.1% under *non-ego* and *ego*, respectively. Similarly, the proportion of full contributions is also close across conditions: 20.5% and 18.1% under *non-ego* and *ego*, respectively (*χ*^2^ test *p*−value = 0.898).

We thus do not find support for H1 in our data. Our conjecture is that, since the scores in Part 1 were relatively high, very few participants chose a null contribution for Part 2. Hence, observing differences between treatments becomes more difficult.

**Fig 3:**
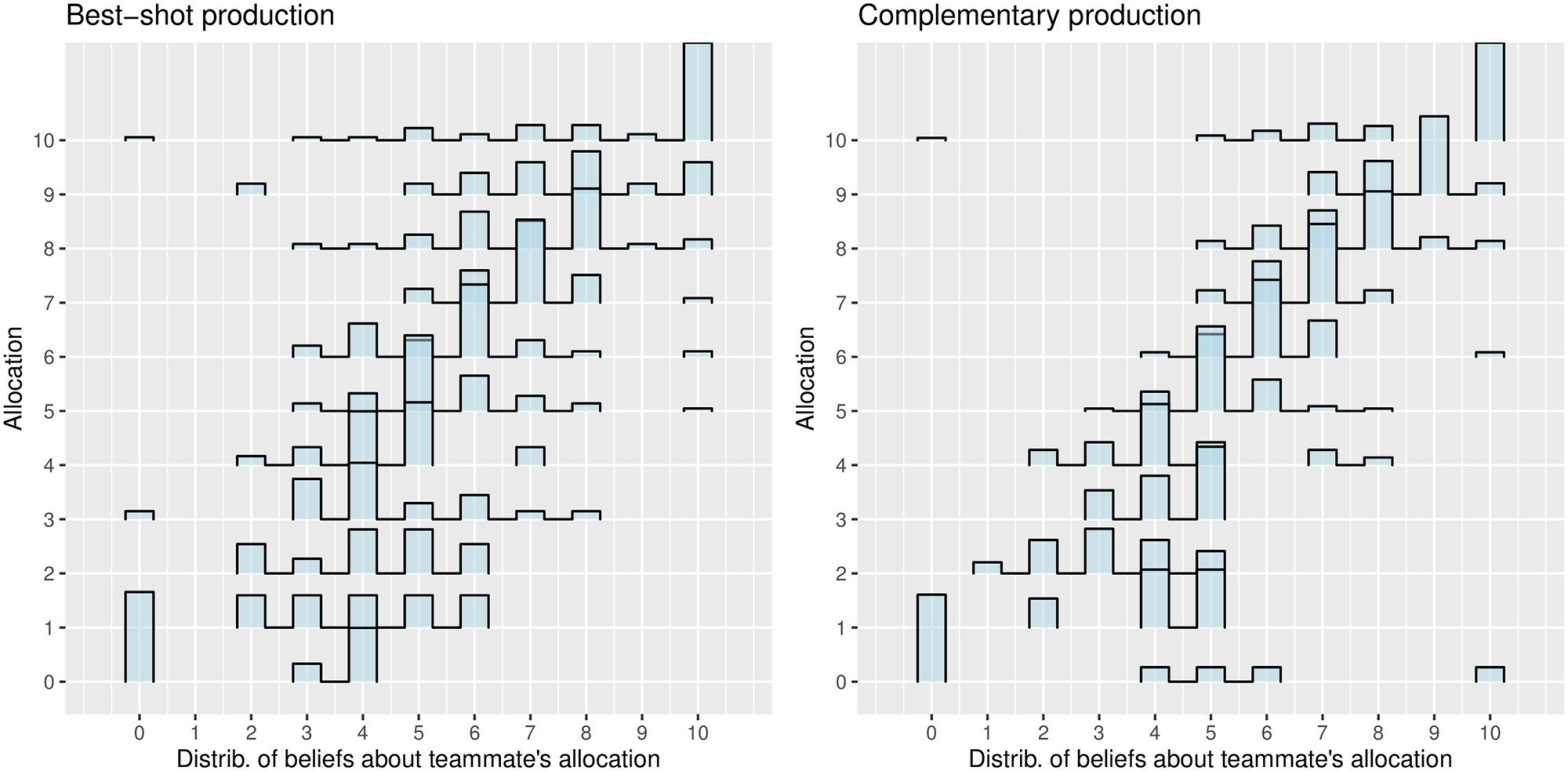
Effort contribution levels conditional on beliefs about teammate’s contribution. Panels correspond to team production functions.

The axes in Fig 3 are analogous to Fig 2. However, the sample is now divided by production functions: *best-shot* on the left and *complementary* on the right. In both panels, the participants seem to select a contribution level similar to the expected teammate’s contribution. The resulting positive slope in both panels provides visual support in favor of H2 and against H3. Regarding H2, most of the participants aim to match the same contribution decision they believe their teammate will make, as in minimum-effort games with multiple coordination equilibria [40]. Regarding H3, we would have expected an alignment around a -45° line, with a high density in the upper-left and bottom-right corners, reflecting the incentives to anti-coordinate. In the next subsection, we will use a regression analysis to validate these hypotheses (and the remaining H4).

### Regression analysis

Table 4 reports the OLS regression results for the predicted contribution decision. We take as covariates the two treatment variables, the beliefs about the teammate’s contribution, their interactions, and the score in Part 1. Results are reported without (cf. columns 1–3) and with (cf. columns 4–6) other control variables. We focus our interpretation on the first three columns, as the other three yield similar results with a minor reduction in statistical significance.

**Table 4:** OLS predictions for the contribution decision.

Dep. Var: Contribution decision	(1)	(2)	(3)	(4)	(5)	(6)
Best-shot	0.0421	1.306^**^	1.325^**^	0.0674	1.304^*^	1.319^**^
	(0.153)	(0.504)	(0.494)	(0.153)	(0.515)	(0.504)
Ego-relevant	-0.178	-0.187	-1.285^**^	-0.117	-0.127	-1.267^**^
	(0.152)	(0.151)	(0.479)	(0.151)	(0.150)	(0.478)
Beliefs (teammate’s contribution)	0.667^***^	0.766^***^	0.692^***^	0.640^***^	0.737^***^	0.659^***^
	(0.0398)	(0.0482)	(0.0606)	(0.0410)	(0.0478)	(0.0604)
Best-shot × Beliefs		-0.202^**^	-0.203^**^		-0.198^*^	-0.199^**^
		(0.0748)	(0.0733)		(0.0768)	(0.0751)
Ego-relevant × Beliefs			0.175^*^			0.182^*^
			(0.0707)			(0.0712)
Score in Part 1	0.366^***^	0.369^***^	0.371^***^	0.315^***^	0.318^***^	0.317^***^
	(0.0520)	(0.0520)	(0.0528)	(0.0605)	(0.0612)	(0.0624)
Constant	-2.395^***^	-3.047^***^	-2.605^***^	-3.507^***^	-4.118^***^	-3.743^***^
	(0.406)	(0.461)	(0.509)	(0.831)	(0.836)	(0.854)
*β*_Beliefs_+ *β*_Best-shot×Beliefs_		0.564	0.489		0.539	0.460
*F*−test (*p*−value)		(<0.001)	(<0.001)		(<0.001)	(<0.001)
Control variables	No	No	No	Yes	Yes	Yes
Observations	590	590	590	586	586	586
R-squared	0.468	0.476	0.483	0.475	0.483	0.489

Additional controls: guess about own score in Part 1, confidence of having a score in top half, gender, age and oneness scale. Robust standard errors in parentheses.

^***^ *p* < 0.001,

^**^ *p* < 0.01,

^*^ *p* < 0.05.

After comparing column 1 with columns 2 and 3, we noted that treatment effects are highly dependent on the beliefs about the teammate’s choice. Without these interactions, the effect of the production function is zero, and the effect of ego-relevance is negative though statistically insignificant. With this in mind, we proceed with the statistical validation of H2 and H3.

#### The interplay of beliefs and contribution decisions across production functions

The validation of H2 and H3 requires focusing on the coefficients for beliefs and its interaction with the *best-shot* variable. The former tells us the change in contributions for one unit increase in the expected teammate’s contribution in the *complementary* treatment. The sum of this coefficient with the interaction term will tell us the analogous reaction for the *best-shot* treatment. According to H2 and H3, the beliefs’ coefficient should be positive, and its sum with the interaction should be negative.

Table 4 reveals that a unit increase in the teammate’s expected contribution increases the participant’s contribution by 0.77 units in the *complementary* treatment. This result provides evidence in favor of H2. On the other hand, and although the interaction term is negative and significant, the effect of a unit increase in the teammate’s expected contribution in the *best-shot* treatment is 0.56 units (see the test for the sum of coefficients at the bottom of Table 4). Hence, this result ratifies the visual evidence against H3.

The experimental evidence for other games with anti-coordination incentives (e.g., battle of the sexes or chicken game) points more often to the predicted behavior [49–52]. Three potential explanations for why we do not observe anti-coordination behavior are: (i) the one-shot nature of the game, (ii) the large action set in our game, and (iii) fairness motives driving efforts to coordinate, against the equilibrium predictions where payoffs in the counter-diagonal maximize the payoff difference.

It is also unclear why participants often choose intermediate activation levels in the *best-shot* treatment. According to our calibration of *ω* based on Part 1’s confidence, an ability greater than *c*/*b* should lead to activating either all or none of the subtasks. One conjecture is that participants assume that subtasks have increasing difficulty. However, if this is the case, most choices should be between 8 and 10 activation units, given their mean number of correctly solved tasks in Part 1 and their accurate beliefs in this direction. Another possibility is that participants’ mixed strategy included not only the extreme actions from an anti-coordination equilibrium (i.e., *A*_*i*_ = 0 or *A*_*i*_ = 10}) but rather a fuller action set. However, since payoffs are larger in the vicinity of the counter-diagonal, the alignment of choices around the 45° diagonal in Fig 3 suggests that this is not the case either. As pointed out by one of the reviewers, the observed choices in the *best-shot* treatment may be a consequence of the difficulties in playing an anti-coordination equilibrium, leading to out-of-equilibrium play that resembles symmetric responses to what they expect from their teammate.

So far, we have evidence against H1 and H3. We did not find that ego-relevance increases the frequency of more extreme contributions. We now detour from the direct validation of hypotheses because column 3 in Table 4 gives an idea about the role of ego-relevance in our setting. We make clear that this is instead an *ex post* analysis that simplifies our report of the evidence for H4, where we explore the interaction effects between treatments.

Returning to Table 4, note that the ego-relevance coefficient is negative and significant, whereas its interaction term with the beliefs is positive and significant. This result suggests that ego-relevance increases responses that mimic conditional cooperation: expectations of high (resp. low) contributions are replied with higher (resp. lower) contributions, compared to these reciprocal responses in the absence of the ego-relevance manipulation. As a robustness check, we report in Table B.1 in S1 Appendix the regression results after excluding participants in the *Non-Ego* treatment that knew beforehand about the Raven matrices task and their use for measuring IQ (11%, 33 out of 302). The results are qualitatively identical.

**Fig 4:**
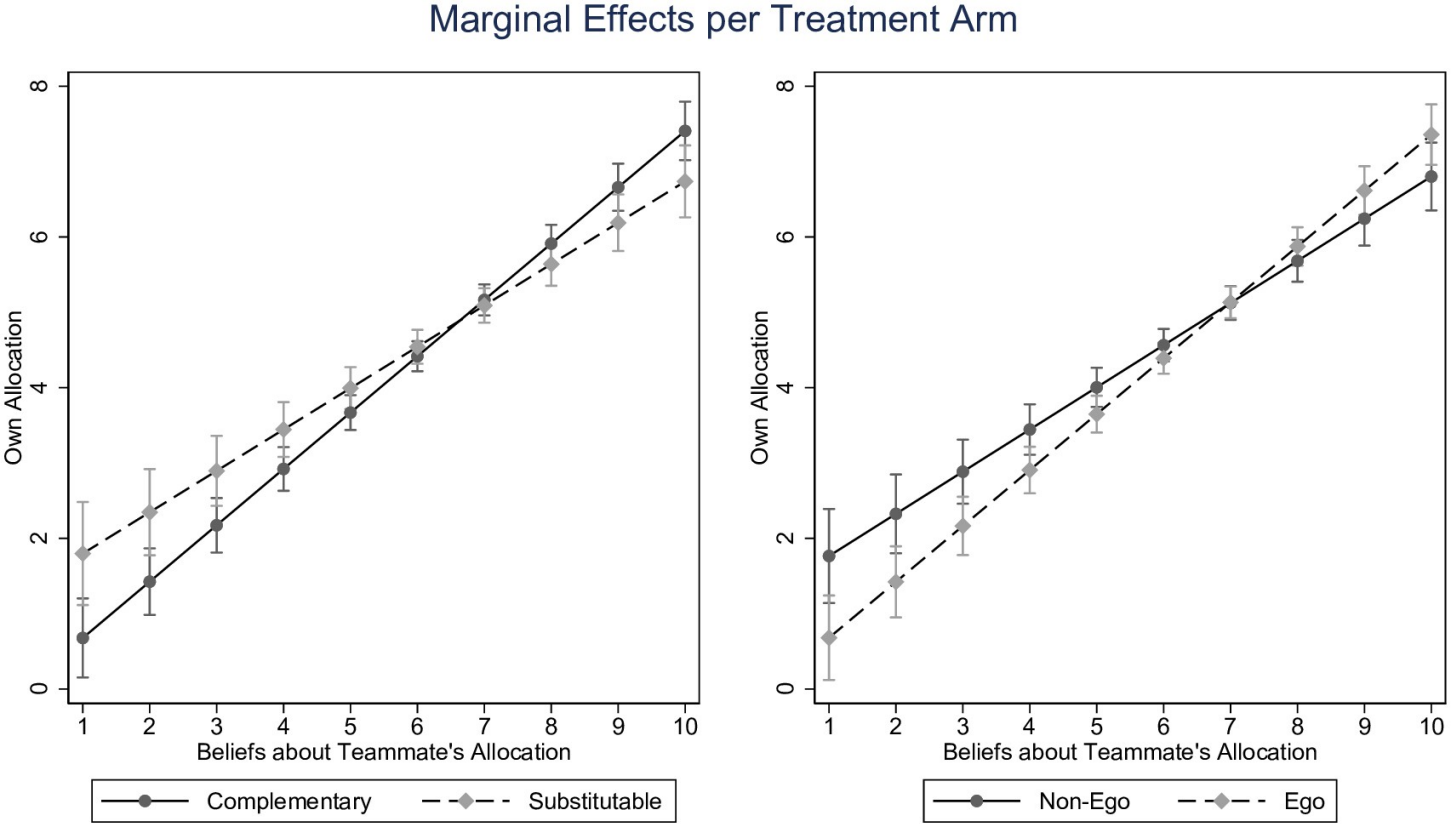
Participants’ contribution decision as a function of the beliefs about teammate’s contribution. Error bars show 95% confidence intervals.

The treatment effects, and their interplay with beliefs, are plotted as marginal effects in Fig 4. The left panel shows that the response to the teammate’s contribution decision is steeper for *complementary* production compared to *best-shot*. On the other hand, the right panel shows a higher steepness for the *ego* condition compared to the *non-ego* condition.

#### Interaction effects between ego-relevance and team production functions

So far, we have separately explored the effects of each treatment arm. In H4, we argue that ego-relevance amplifies the response to the beliefs regarding the teammate’s contribution, and the effect is differential across production functions. Hence, for testing H4, we need to include triple interactions between our two treatment variables and the participant’s beliefs. Since the coefficients from triple interactions are not straightforward to interpret, we adopt a methodologically equivalent but more tractable strategy using a Chow-like test. Intuitively, we split our sample according to a treatment variable (i.e., ego-relevance in models 1 and 2) and interact the other treatment variable (i.e., *best-shot*) with beliefs. The triple interaction is statistically significant if we reject the hypothesis of equality of coefficients across the two regressions, according to a Chow test.

The results of this exercise are reported in Table 5. The *p*−values of the reported Chow tests reveal that beliefs do not moderate interaction effects. Table B.2 in S1 Appendix, an expanded version of Table 4 including triple interactions, validates this result and shows that the interaction between the two treatments was not significant either. This evidence points in the direction that the two treatment effects operate independently, leaving H4 without empirical support. One explanation for the absence of interaction effects is that the treatment differences were capturing the steepness in the reaction function to the beliefs about the teammate’s contribution. Since participants behaved as conditional contributors with both production functions, it was very hard for the triple interaction to detect small changes in this slope.

**Table 5:** OLS results for combined effects of ego-relevance, team production function and beliefs about teammate’s contributions. *p*−values corresponding to tests for seemingly unrelated estimations reported in brackets next to the Chow test p-values.

VARIABLES	Dependent variable: Contribution decision			
	Selected subsample			
	Non-ego	Ego	Complementary	Best-Shot
	(1)	(2)	(3)	(4)
Best-shot	1.448	1.085		
	(0.749)	(0.646)		
Ego-relevant			-1.250*	-1.359
			(0.608)	(0.752)
Beliefs (about teammate’s allocation)	0.662^***^	0.827^***^	0.678^***^	0.433^***^
	(0.0702)	(0.0605)	(0.0671)	(0.0892)
Best-shot × Beliefs	-0.214	-0.165		
	(0.113)	(0.0951)		
Ego-relevant × Beliefs			0.179*	0.195
			(0.0891)	(0.113)
Constant	-3.772^***^	-4.808^***^	-3.156^***^	-3.013*
	(1.109)	(1.241)	(1.126)	(1.261)
Chow tests. *p*−values in squared brackets []				
Best-shot (1) vs. (2)	0.14	[0.709]		
Best-shot × Beliefs (1) vs. (2)	0.12	[0.731]		
Ego-relevant (3) vs. (4)			0.01	[0.909]
Ego-relevant × Beliefs (3) vs. (4)			0.01	[0.913]
Observations	300	286	294	292
R-squared	0.446	0.543	0.568	0.420

Additional controls in all models: guess about own score in Part 1, confidence of having a score in top half, gender, age and oneness scale. Robust standard errors in parentheses.

^***^ *p* < 0.001,

^**^ *p* < 0.01,

^*^ *p* < 0.05.

## Concluding discussion

We designed and conducted an online experiment to understand how ego-relevance affects individual contributions to a team production task. We devised two different technologies for team production. Under *best-shot*, participants have theoretical incentives to “anti-coordinate” their contributions since the team production depends on the maximum individual performance. Under *complementary*, participants have incentives to coordinate on an identical contribution since the team production has the structure of a minimum-effort game. The introduction of ego-relevance alters the ability threshold they expect from their (symmetric) teammate to opt for a positive contribution. Moreover, we predict that the best-response to their teammate’s contribution effort becomes steeper with ego-relevance.

Participants often select a contribution level that matches their teammate’s expected contribution. This behavior matches the predicted coordination equilibria under *complementary* production. Similar (though noisier) behavior is observed under the *best-shot* production function. Nevertheless, in the latter case, this result drives behavior away from the predicted anti-coordination equilibria. Other games involving these asymmetric outcomes are more successful, perhaps due to their repeated nature and more compact action set [49, 51].

We also find that ego-relevance makes the reaction to the teammate’s contribution steeper. Following Fig 2, ego-relevance increases the chances of mirroring the expected teammate’s contribution. Our model does not account for this result because we predicted that the main effect of ego-relevance was to alter the threshold leading to a null contribution equilibrium. A behavioral *ex post* conjecture is that ego-relevance raises the salience of symmetry in the contribution decision. As teams are relatively homogeneous in their ability, ego-relevance seems to help the participants mimic their teammate. This pattern is not necessarily a “good” behavior from the perspective of resource efficiency because it neglects the team production function. Team tasks resembling our *best-shot* structure would be negatively affected if team members aim to mirror their mates.

The effect of ego-relevance, yielding steeper reactions in own effort to the expectations of teammates’ effort, has implications for organizational decision-making in knowledge-based industries (e.g., the education sector and creative industries). When sorting workers into homogeneous groups (the scenario emulated in our setting), a potential concern is whether talent will be genuinely complementary or whether “superstar effects” will eclipse collaboration. We have learned that, even when monetary incentives point to the latter, participants tend not to choose extreme individual contribution levels, revealing that they perceive some complementarity in their efforts. Thus, ego-relevance fosters that mirroring efforts among workers. Moreover, managers can also benefit from workers’ ego concerns to reframe team tasks that are not too difficult and trigger collaboration. For complex tasks, ego-protection motives may backfire, though our study does not contribute with evidence in this direction. However, a recent paper studying team effort decisions in engaging versus menial tasks has indeed found that individuals tend to shirk more in engaging tasks [53].

A caveat in our experimental design regarding external validity is the homogeneity in teams’ ability. We opted for pairing participants with similar abilities to simplify our predictions, aiming to understand the ego-relevance of the task without confounding this effect with other ego-utility factors associated with heterogeneity in abilities. However, it is entirely plausible that the nature of the anti-coordination equilibria would be better understood under such heterogeneities. Moreover, endogenous teams embedded in a *best-shot* production structure are probably heterogeneous in abilities, giving more room for anti-coordination equilibria to emerge. In the same line, a heterogeneous sorting of teammates’ abilities could have led to a stronger manipulation of ego-relevance. This more intense ego manipulation might have revealed interaction effects between treatments that we do not observe but can be explored in further research.

## Supporting information

S1 Appendix (PDF)

## References

[1] Alchian A. A. and Demsetz H. (1972). Production, information costs, and economic organization. *The American Economic Review*, 62(5): 777–795.

[2] Büyükboyacı M. and Robbett A. (2017). Collaboration and free-riding in team contests. Labour Economics, 49: 162–178. doi: 10.1016/j.labeco.2017.11.001

[3] Bénabou R. and Tirole J. (2002). Self-confidence and personal motivation. *The Quarterly Journal of Economics*, 117(3): 871–915. doi: 10.1162/003355302760193913

[4] Bénabou R. and Tirole J. (2003). Intrinsic and extrinsic motivation. *The Review of Economic Studies*, 70(3): 489–520. doi: 10.1111/1467-937X.00253

[5] Köszegi B. (2006). Ego utility, overconfidence, and task choice. *Journal of the European Economic Association*, 4(4): 673–707. doi: 10.1162/JEEA.2006.4.4.673

[6] Thompson T., Davidson J. A., and Barber J. G. (1995). Self-worth protection in achievement motivation: Performance effects and attributional behavior. *Journal of Educational Psychology*, 87(4): 598. doi: 10.1037/0022-0663.87.4.598

[7] Tice D. M. (1991). Esteem protection or enhancement? self-handicapping motives and attributions differ by trait self-esteem. *Journal of Personality and Social Psychology*, 60(5): 711. doi: 10.1037/0022-3514.60.5.711

[8] Barron K. and Gravert C. (2022). Confidence and career choices: An experiment. *The Scandinavian Journal of Economics*, 124(1): 35–68. doi: 10.1111/sjoe.12444

[9] Fischer M. and Sliwka D. (2018). Confidence in knowledge or confidence in the ability to learn: An experiment on the causal effects of beliefs on motivation. Games and Economic Behavior, 111: 122–142. doi: 10.1016/j.geb.2018.02.005

[10] Mertins V. and Hoffeld W. (2015). Do overconfident workers cooperate less? the relationship between overconfidence and cooperation in team production. *Managerial and Decision Economics*, 36(4): 265–274. doi: 10.1002/mde.2667

[11] Tsai C. I. and Xie J. L. (2017). How incidental confidence influences self-interested behaviors: A double-edged sword. *Journal of Behavioral Decision Making*, 30(5): 1168–1181. doi: 10.1002/bdm.2032

[12] Castagnetti A. and Schmacker R. (2022). Protecting the ego: Motivated information selection and updating. European Economic Review, 142: 104007. doi: 10.1016/j.euroecorev.2021.104007

[13] Hoffman M. (2016). How is information valued? evidence from framed field experiments. *The Economic Journal*, 126(595): 1884–1911. doi: 10.1111/ecoj.12401

[14] Fischbacher U., Gächter S., and Fehr E. (2001). Are people conditionally cooperative? evidence from a public goods experiment. *Economics Letters*, 71(3): 397–404. doi: 10.1016/S0165-1765(01)00394-9

[15] Fischbacher U. and Gächter S. (2010). Social preferences, beliefs, and the dynamics of free riding in public goods experiments. *American Economic Review*, 100(1): 541–56. doi: 10.1257/aer.100.1.541

[16] Gächter S., Kölle F., and Quercia S. (2017). Reciprocity and the tragedies of maintaining and providing the commons. *Nature Human Behaviour*, 1(9): 650–656. doi: 10.1038/s41562-017-0191-5 28944297 PMC5604734

[17] Ellingsen T., Johannesson M., Mollerstrom J., and Munkhammar S. (2012). Social framing effects: Preferences or beliefs? *Games and Economic Behavior*, 76(1): 117–130. doi: 10.1016/j.geb.2012.05.007

[18] Guerra G. and Zizzo D. J. (2004). Trust responsiveness and beliefs. *Journal of Economic Behavior & Organization*, 55(1): 25–30. doi: 10.1016/j.jebo.2003.03.003

[19] Neumann T. and Vogt B. (2009). Do players’ beliefs or risk attitudes determine: The equilibrium selections in 2x2 coordination games? *Working Paper Series*.

[20] Mehta J., Starmer C., and Sugden R. (1994). The nature of salience: An experimental investigation of pure coordination games. *The American Economic Review*, 84(3): 658–673.

[21] Chaudhuri A., Paichayontvijit T., and So T. (2015). Team versus individual behavior in the minimum effort coordination game. Journal of Economic Psychology, 47: 85–102. doi: 10.1016/j.joep.2015.02.002

[22] Dong L., Montero M., and Possajennikov A. (2018). Communication, leadership and coordination failure. *Theory and decision*, 84(4): 557–584. doi: 10.1007/s11238-017-9617-9

[23] Heger S. A. and Papageorge N. W. (2018). We should totally open a restaurant: How optimism and overconfidence affect beliefs. Journal of Economic Psychology, 67: 177–190. doi: 10.1016/j.joep.2018.06.006

[24] Coutts A. (2019). Good news and bad news are still news: Experimental evidence on belief updating. *Experimental Economics*, 22(2): 369–395. doi: 10.1007/s10683-018-9572-5

[25] Ertac S. (2011). Does self-relevance affect information processing? experimental evidence on the response to performance and non-performance feedback. *Journal of Economic Behavior & Organization*, 80(3): 532–545. doi: 10.1016/j.jebo.2011.05.012

[26] Eil D. and Rao J. M. (2011). The good news-bad news effect: asymmetric processing of objective information about yourself. *American Economic Journal: Microeconomics*, 3(2): 114–38.

[27] Mobius, M. M., Niederle, M., Niehaus, P., and Rosenblat, T. S. (2014). Managing self-confidence: Theory and experimental evidence. Technical report, National Bureau of Economic Research.

[28] Buser T., Gerhards L., and Van Der Weele J. (2018). Responsiveness to feedback as a personal trait. *Journal of Risk and Uncertainty*, 56(2): 165–192. doi: 10.1007/s11166-018-9277-3 31007385 PMC6445505

[29] Grossman Z. and Owens D. (2012). An unlucky feeling: Overconfidence and noisy feedback. *Journal of Economic Behavior & Organization*, 84(2): 510–524. doi: 10.1016/j.jebo.2012.08.006

[30] Drobner, C. and Goerg, S. (2021) Motivated belief updating and rationalization of information *Kiel*, *Hamburg: ZBW-Leibniz Information Centre for Economics*

[31] Sebald A. and Walzl M. (2015). Optimal contracts based on subjective performance evaluations and reciprocity. Journal of Economic Psychology, 47: 62–76. doi: 10.1016/j.joep.2015.01.004

[32] Szymanski S. (2003). The economic design of sporting contests. *Journal of Economic Literature*, 41(4): 1137–1187. doi: 10.1257/jel.41.4.1137

[33] Franck E. and Nüesch S. (2010). The effect of talent disparity on team productivity in soccer. *Journal of Economic Psychology*, 31(2): 218–229. doi: 10.1016/j.joep.2009.12.003

[34] Chapsal A. and Vilain J.-B. (2019). Individual contribution in team contests. Journal of Economic Psychology, 75: 102087. doi: 10.1016/j.joep.2018.07.003

[35] Brookins P., Lightle J. P., and Ryvkin D. (2015). An experimental study of sorting in group contests. Labour Economics, 35: 16–25. doi: 10.1016/j.labeco.2015.03.011

[36] Brookins P., Lightle J. P., and Ryvkin D. (2018). Sorting and communication in weak-link group contests. Journal of Economic Behavior & Organization, 152: 64–80. doi: 10.1016/j.jebo.2018.05.010

[37] Sheremeta R. M. (2011). Perfect-substitutes, best-shot, and weakest-link contests between groups. Korean Economic Review, 27: 5–32.

[38] Kremer M. (1993). The o-ring theory of economic development. *The Quarterly Journal of Economics*, 108(3): 551–575. doi: 10.2307/2118400

[39] Rosen S. (1981). The economics of superstars. *The American Economic Review*, 71(5): 845–858.

[40] Van Huyck J. B., Battalio R. C., and Beil R. O. (1990). Tacit coordination games, strategic uncertainty, and coordination failure. *The American Economic Review*, 80(1): 234–248.

[41] Raven, J. and Raven, J. (2003). Raven progressive matrices. In *Handbook of nonverbal assessment*, pages 223–237. Springer.

[42] Trautmann S. T. and van de Kuilen G. (2015). Belief elicitation: A horse race among truth serums. *The Economic Journal*, 125(589): 2116–2135. doi: 10.1111/ecoj.12160

[43] Danz D., Vesterlund L., and Wilson A. J. (2022). Belief elicitation and behavioral incentive compatibility. *The American Economic Review*. doi: 10.1257/aer.20201248

[44] Dutcher, G., Salmon, T., and Saral, K. J. (2015). Is’ real’effort more real? *Available at SSRN 2701793*.

[45] Gächter S., Huang L., and Sefton M. (2016). Combining “real effort” with induced effort costs: the ball-catching task. *Experimental Economics*, 19(4): 687–712. doi: 10.1007/s10683-015-9465-9 28035190 PMC5153668

[46] Gächter S., Starmer C., and Tufano F. (2015). Measuring the closeness of relationships: a comprehensive evaluation of the’inclusion of the other in the self’scale. *PLoS one*, 10(6): e0129478. doi: 10.1371/journal.pone.0129478 26068873 PMC4466912

[47] Bergman L. R., Ferrer-Wreder L., and Žukauskienė R. (2015). Career outcomes of adolescents with below average IQ: Who succeeded against the odds? Intelligence, 52: 9–17. doi: 10.1016/j.intell.2015.06.003

[48] Chen D.L., Schonger M., and Wickens C. (2016). oTree-an open-source platform for laboratory, online, and field experiments. Journal of Behavioral and Experimental Finance, 9: 88–97. doi: 10.1016/j.jbef.2015.12.001

[49] Bornstein G., Budescu D., and Zamir S. (1997). Cooperation in intergroup, n-person, and two-person games of chicken. *Journal of Conflict Resolution*, 41(3): 384–406. doi: 10.1177/0022002797041003003

[50] Cooper R., DeJong D. V., Forsythe R., and Ross T. W. (1989). Communication in the battle of the sexes game: some experimental results. *The RAND Journal of Economics*, pages 568–587. doi: 10.2307/2555734

[51] Wit A. P. and Wilke H. A. (1992). The effect of social categorization on cooperation in three types of social dilemmas. *Journal of Economic Psychology*, 13(1): 135–151. doi: 10.1016/0167-4870(92)90056-D

[52] Zizzo D. J. and Tan J. H. (2007). Perceived harmony, similarity and cooperation in 2 × 2 games: an experimental study. *Journal of Economic Psychology*, 28(3): 365–386. doi: 10.1016/j.joep.2006.06.008

[53] Isoni A. and Eubanks D. (2022). The effect of relative performance feedback on effort and free riding in repetitive and engaging team tasks. *Working Paper*.

