# Supplementary material for: Ego-relevance in team production
Source: PLoS One. 2022 Dec 21;17(12):e0279391. doi: 10.1371/journal.pone.0279391 (PMC9770434; doi:10.1371/journal.pone.0279391)
Supplement: S1 Appendix — (PDF) [file pone.0279391.s001.pdf]

Ego-relevance in team production: Appendix

César Mantilla[1] **and Zahra Murad**[2]

The replication files can be downloaded from: `10.17632/7mfdv9dr2x.1`

A Evidence that our treatment induced ego-relevance

Table 3 shows that participants in the *Ego-Relevant* condition were more likely to pay and receive feedback on their scores from Part 1, 2, or both. Fig A.1 confirms this finding, revealing that most of the additional share of participants paying to learn their scores in the *ego* treatment did so for Parts 1 and 2.

We use a probit model to validate that the ego-relevance manipulation was successful and to explore whether the decision to pay for this feedback is moderated by the participants' self-esteem. The dependent variable is equal to 1 when the participant paid to reveal her score in Part 1, 2, or both; and zero otherwise. As a proxy for initial confidence levels about task performance, we employ, in different specifications, the participants' guess regarding their absolute score, and their confidence in scoring in the top half and the top quarter among all participants (Tice, 1991).

Table A.1 reports the marginal effects of this probit model. The *ego* condition increases probability to pay for this feedback on performance by 13 percentage points. The variables capturing confidence in initial performance have negative coefficients, and they are significant in models (1) and (2), suggesting that more confident participants are less likely to pay and learn their scores. This result is partly aligned with Tice's (1991) hypothesis that prior self-esteem affects self-handicapping behaviour.

[1]Department of Economics, Universidad del Rosario.

[2]Economics and Finance, University of Portsmouth, Portsmouth. UNEC Cognitive Economics Center, Azerbaijan State University of Economics, Azerbaijan.

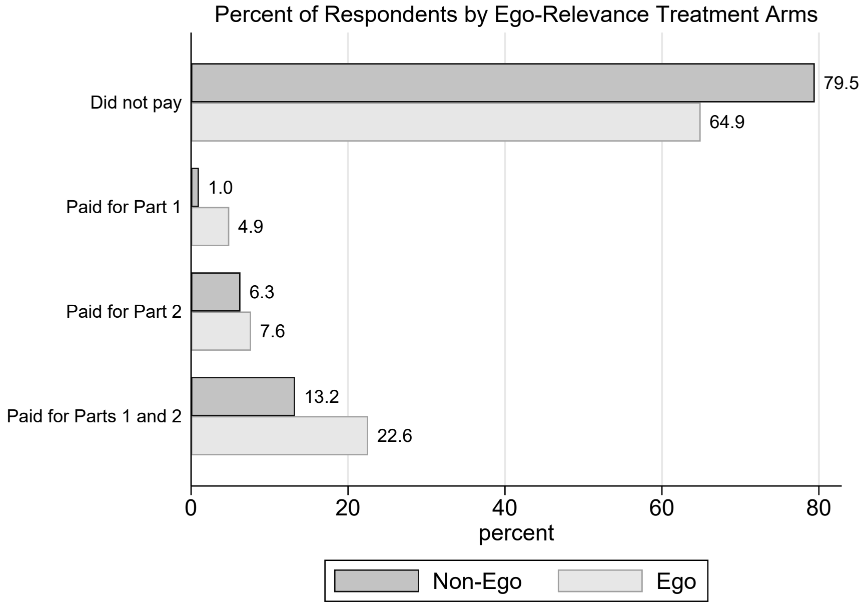

Figure A.1: Distribution of payment decisions for learning the scores in Parts 1 and 2 at the end of the game. Each score revelation costs £0.10.

VARIABLES	Paid to reveal at least one score		
	(1)	(2)	(3)
Ego-Relevant	0.133***	0.132***	0.132***
	(0.0362)	(0.0363)	(0.0364)
Above median: Guess correct response	-0.0795*		
	(0.0402)		
Above median: Belief Top 50%		-0.0814*	
		(0.0381)	
Above median: Belief Top 25%			-0.0468
			(0.0379)
Observations	586	586	586

Additional controls: Performance in Part 1, gender, age and oneness scale. Robust standard errors in parentheses. *** $p < 0.001$, ** $p < 0.01$, * $p < 0.05$.

Table A.1: Marginal effects from probit model for the decision to pay and learn at least one score

B Additional Figures and Tables

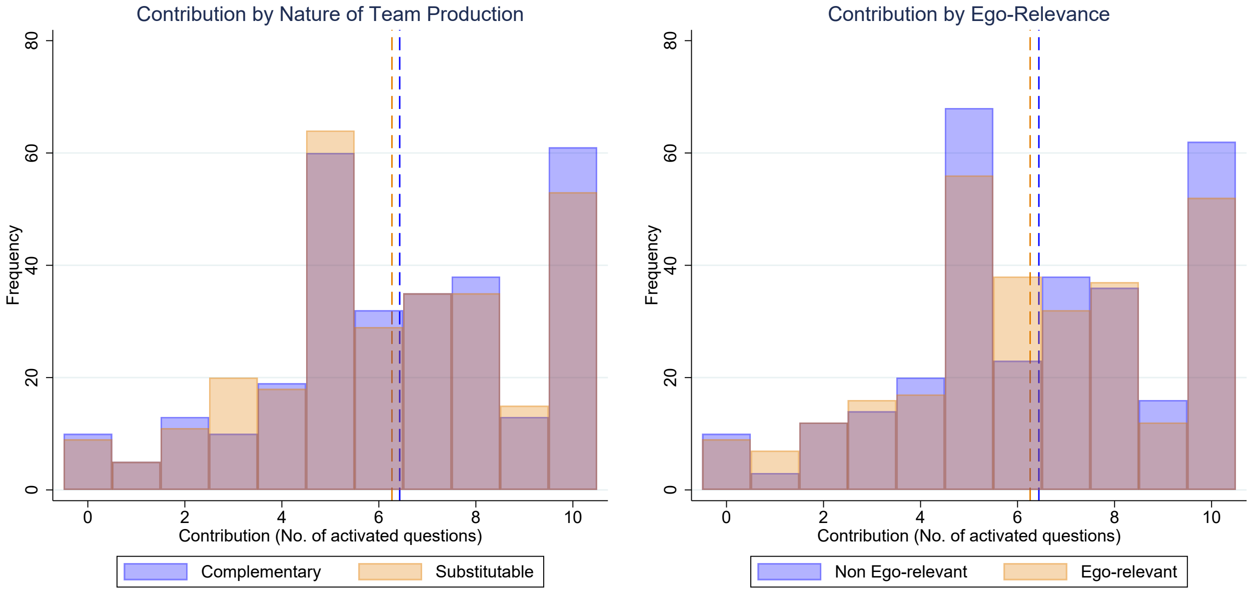

Figure B.1: Distribution of contribution decisions by the team production function (left panel) and ego-relevance (right panel). The dashed vertical lines correspond to the average contribution per treatment variation.

Dep. Var: Contribution decision	(1)	(2)	(3)	(4)	(5)	(6)
Best-shot	-0.0300	1.129*	1.132*	0.00131	1.154*	1.151*
	(0.157)	(0.448)	(0.448)	(0.157)	(0.464)	(0.464)
Ego-relevant	-0.0964	-0.0948	-1.058*	-0.0374	-0.0359	-1.040*
	(0.156)	(0.155)	(0.445)	(0.156)	(0.156)	(0.448)
Beliefs (teammate's contribution)	0.719***	0.806***	0.741***	0.689***	0.776***	0.708***
	(0.0354)	(0.0375)	(0.0481)	(0.0374)	(0.0391)	(0.0499)
Best-shot x Beliefs		-0.182**	-0.182**		-0.181**	-0.181**
		(0.0671)	(0.0668)		(0.0699)	(0.0696)
Ego-relevant x Beliefs			0.151*			0.158*
			(0.0658)			(0.0665)
Score in Part 1	0.345***	0.345***	0.345***	0.274***	0.274***	0.271***
	(0.0508)	(0.0502)	(0.0504)	(0.0572)	(0.0566)	(0.0565)
Constant	-2.623***	-3.193***	-2.775***	-3.622***	-4.191***	-3.812***
	(0.413)	(0.457)	(0.486)	(0.867)	(0.882)	(0.908)
Control variables	No	No	No	Yes	Yes	Yes
Observations	511	511	511	507	507	507
R-squared	0.515	0.522	0.527	0.521	0.528	0.533

Additional controls: guess about own score in Part 1, confidence of having a score in top half, gender, age and oneness scale. Robust standard errors in parentheses. *** $p < 0.001$, ** $p < 0.01$, * $p < 0.05$.

Table B.1: OLS results for the determinants of the allocation decision excluding participants in the *Non-Ego* treatment that knew about the Raven matrices task (33 out of 303).

Dep. Var: Contribution decision	(1)	(2)	(3)	(4)	(5)	(6)
Best-shot	0.0941	1.344*	1.332	0.138	1.353*	1.422
	(0.220)	(0.521)	(0.721)	(0.222)	(0.530)	(0.733)
Ego-relevant	-0.125	-1.261*	-1.276*	-0.0450	-1.224*	-1.141
	(0.199)	(0.496)	(0.603)	(0.201)	(0.494)	(0.610)
Best-shot x Ego-relevant	-0.107	-0.0431	-0.0132	-0.145	-0.0744	-0.239
	(0.304)	(0.299)	(0.952)	(0.304)	(0.299)	(0.966)
Beliefs (teammate's contribution)	0.667***	0.692***	0.691***	0.640***	0.659***	0.664***
	(0.0399)	(0.0607)	(0.0688)	(0.0411)	(0.0605)	(0.0687)
Best-shot x Beliefs		-0.203**	-0.201		-0.198**	-0.210
		(0.0733)	(0.107)		(0.0751)	(0.110)
Ego-relevant x Beliefs		0.175*	0.177*		0.181*	0.168
		(0.0708)	(0.0882)		(0.0712)	(0.0902)
Best-shot x Ego-relevant x Beliefs			-0.00477			0.0263
			(0.141)			(0.144)
Score in Part 1	0.366***	0.371***	0.371***	0.316***	0.317***	0.316***
	(0.0520)	(0.0529)	(0.0527)	(0.0606)	(0.0625)	(0.0625)
Constant	-2.422***	-2.616***	-2.611***	-3.543***	-3.762***	-3.793***
	(0.412)	(0.515)	(0.560)	(0.832)	(0.856)	(0.879)
Observations	590	590	590	586	586	586
R-squared	0.468	0.483	0.483	0.475	0.489	0.489

Additional controls: guess about own score in Part 1, confidence of having a score in top half, gender, age and oneness scale. Robust standard errors in parentheses. *** $p < 0.001$, ** $p < 0.01$, * $p < 0.05$.

Table B.2: OLS predictions for the contribution decision, including triple interaction effects between treatments and beliefs.

C Protocol

Consent form

Please read the consent form below and press the continue button if you agree to each bullet point.

- I voluntarily agree to take part in this study.
- I have been given a full instruction by the investigators of the nature, purpose, location and likely duration of the study, and of what I will be expected to do.
- I consent to my data that I provide in this experiment being used for this study. I understand that all personal data relating to volunteers is anonymised and held and processed in the strictest confidence. I understand that the research data will be used for producing a research article to be published in scientific journals. The anonymised data may be published as an open-access data source.
- I understand that I am free to withdraw from the study at any time during the experiment without needing to justify my decision and without prejudice. I also understand that if I withdraw from the study once the study is finalised I can ask for my data to be deleted by providing my Prolific ID.
- I acknowledge that in consideration for completing the study I shall receive earnings at the end of the study. I recognise that I will receive £2 plus additional payment that will depend on my and other participants' decisions as explained in the instructions for the experiment.
- I confirm that I have read and understood the above and freely consent to participating in this study. I have been given adequate time to consider my participation and agree to comply with the instructions and restrictions of the study.

Instructions

Welcome to our study! You will receive £2 for participating in this study. Plus you can earn additional money depending on your decisions during the study. Please read and follow the instructions carefully. They contain everything you need to know.

Part 1: ***IQ Task/ Pattern Task***

```
Note 1: treatment variations are displayed in italics.
Note 2: this paragraph appears only in the ego-relevance treatment.
```

The Part 1 tasks are taken from an Intelligence Quotient (IQ) test that is commonly used to measure people's intelligence levels. Previous research has shown that people scoring high in IQ tests have been found to get higher salaries, obtain better job position and report higher satisfaction with their lives.

You will be shown 10 patterns with a missing element. Your task is to select the option that completes the pattern from several options given at the bottom of the screen. An example pattern is provided below, where option 4 is the correct answer.

You will have 2 minutes to complete a set of 10 patterns. Each correct answer will add 1 point to your score and wrong answers will not affect your score.

Your score does not increase your payment in Part 1, but in Part 2 you will be matched to a teammate with the closest score to yours. This may determine your payments in Part 2 of the Study. So it is in your interest to score as high as you can.

Everyone else will complete the same set of *IQ/Pattern* tasks.

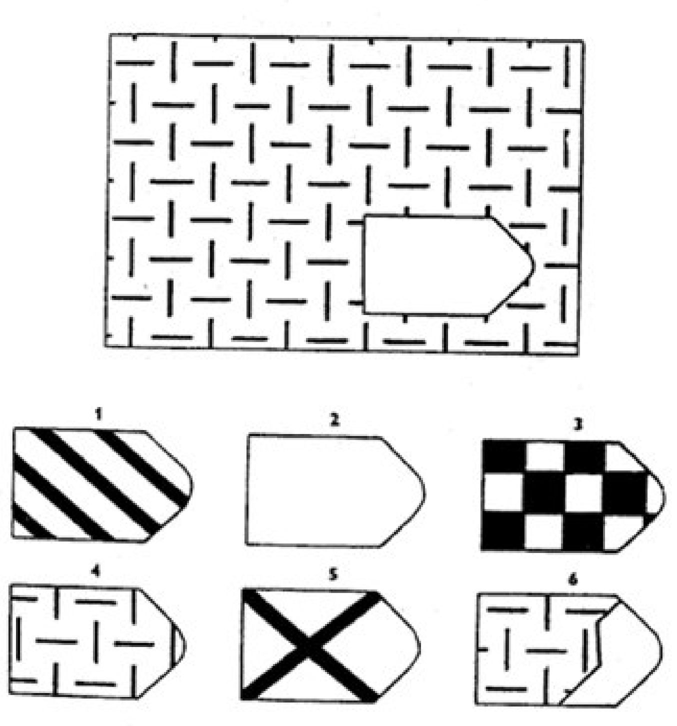

Please click Continue to start the *IQ/Pattern* Task.

Execution of Part 1's Raven Task

Belief elicitation after completing Part 1's Task

Thanks for completing the *IQ/Pattern* Task.

1. Please tell us how many tasks out of 10 you think you got right?
2. Please tell us how confident you are that your score is in the top half of all other scores of all participants in this study by moving the slider below. That is, if we ranked all scores from highest to lowest, how confident are you that your rank would be in the top 50% of all scores?

(If you are completely sure your score is in the top half, choose 100% confident. If you are completely sure your score is not in the top half, then choose 0% confident. Choose intermediary values if you are uncertain whether your score is in the top or bottom half of all scores. Please try to express your confidence in scoring in the top half as accurately as you can.)

3. Also tell us how confident you are that your score is in the top quarter of all other scores by moving the slider below. That is if we ranked all scores from highest to lowest, how confident are you that your rank would be in the top 25% of all scores.

(If you are completely sure your score is in the top quarter, choose 100% confident. If you are completely sure your score is not in the top quarter, then choose 0% confident. Choose intermediary values if you are uncertain whether your score is in the top quarter of all scores. Please try to express your confidence of scoring in the top quarter as accurately as you can.)

Team Decision: *[Complementary/Substitutable Team Production Manipulation]*

Your scores in previous *IQ/Pattern* Task were calculated. We will rank all 20 participants (including you) depending on the scores in the Task. Depending on your score and your rank, you will be matched with another person as shown below to form a Team. We will know your scores, but unfortunately, you will not learn what score you or your teammate achieved or who your teammate is at any point of the study.

In this part of the experiment, you will have to complete a new set of 10 *IQ/Pattern* Tasks in 2 minutes. Your earnings will depend on your and your teammate's decisions and scores.

You are about to answer a new set of 10 *IQ/Pattern* Tasks. You have to decide how many, but not which ones, of the Tasks will be used to compute your payment. We call them the ACTIVATED answers.

You are now given an additional £1.00 that you can spend to activate your answers from the *IQ/Pattern* Task. Activating each answer costs £0.10.

The software will randomly select the number of ACTIVATED answers out of the 10 *IQ/Pattern* Tasks to calculate your ACTIVATED score.

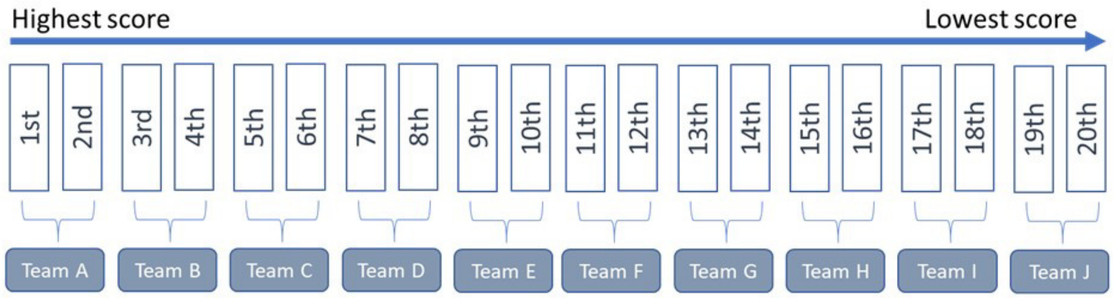

YOUR ACTIVATED SCORE, TEAMMATE ACTIVATED SCORE and TEAMSCORE

Your additional earnings from this study depend on your TEAMSCORE.

The TEAMSCORE will be the *MINIMUM/MAXIMUM* between your own and your teammate's ACTIVATED score. That is, your TEAMSCORE depends on the *MINIMUM/MAXIMUM* number of correct answers you and your teammate got among the ACTIVATED answers.

TEAMSCORE = *Minimum/Maximum* (Your Activated Score; Teammate Activated Score)

You and your teammate will simultaneously decide how many answers to activate. It means that both you and your teammate will earn the same amount from working on IQ/Pattern task, but your total activation costs may differ depending on how many questions each of you decided to activate. We will subtract your activation cost from the additional £1.00 that you are given in this part.

Your earnings

To calculate your earnings, we will multiply your TEAMSCORE by £0.25 and subtract your cost of activating the questions. Your additional earnings will be equal to £1 that we give you to activate answers minus the £0.10 multiplied with the number you choose to activate plus your TEAMSCORE multiplied with £0.25. The formula for this is as below:

Additional Earnings = £1.00 - £0.10 × ACTIVATED ANSWERS + £0.25×TEAMSCORE

At the end of the study, you will learn about your additional earnings, but you will not learn what your and your teammate's scores were or how many answers your teammate activated.

Let's look at an example to better understand how your earning will be calculated.

Example 1

You and your teammate decide to activate all 10 of the answers. Since you have both activated all 10 answers, the software will look at the correctness of all your answers. Suppose YOUR

ACTIVATED SCORE was 9 questions right and your TEAMMATE's ACTIVATED SCORE was 7 questions right. Then the TEAMSCORE will be *9/7*, since *9/7* was the *MAXIMUM/MINIMUM* score between you and your teammate ACTIVATED score. Your and your teammate's earnings will thus be: £1.00 - £0.10×10 + £0.25 × *9/7* = *£2.25/1.75*.

Example 2

You have chosen to activate 7 answers and your teammate have chosen to activate 4 answers. The software randomly activates 7 out of the 10 answers from the set of 10 *IQ/Pattern* tasks, and then checks how many are correct. The software randomly activates 4 out of the 10 answers of your teammate from the set of 10 *IQ/Pattern* tasks, and then checks how many are correct. Suppose your ACTIVATED SCORE is 6 of 7 answers. Suppose your TEAMMATES' ACTIVATED SCORE is 2 of 4 answers.

Then the TEAMSCORE will be *6/2*, since *6/2* was the MAXIMUM/MINIMUM number of correct answers between you and your teammate. Your final earnings will be £1.00 + £0.25× *6/2* - £0.10×7 = *£1.80/0.80* and your teammate's earnings will be £1.00 + £0.25 × *6/2* –£0.10×4= *£2.10/1.10*.

You will only learn about your final earnings, but not about your or your teammate's correct answers.

Quiz [Correct answers in bold]

To make sure you understood how your earnings will be calculated, please answer the following quiz on your screen. You will not be able to proceed to the study unless you can answer all questions.

Q1: You activated 4 answers and your teammate activated 0 answers. You answered correctly 3 out of the 4 randomly selected questions.

Select the correct answer:

- Your activated score will be: **a) 3** b) 4 c) 5 d) 0
- Your teammate activated score will be: a) 3 b) 4 c) 5 **d) 0**
- Your team score will be: **a) 3**, b) 4, c) 5, **d) 0**
- Your earnings will be: **a) less than your teammate's earnings,** b) larger than your teammate earnings, c) equal to your teammate earnings.
- YOUR ACTIVATED SCORE: **[3]**

- TEAMMATE'S ACTIVATED SCORE: **[0]**
- TEAMSCORE: **[3/0]**
- Your earnings: **[£1.35/£0.60]**
- Your teammate's earnings **[£1.75/£1.00]**

Q2: You and your teammate activated 6 answers each. Suppose you have answered 5 of the 6 randomly selected questions correctly, while your teammate answered 6 out of 6 questions correctly.

Select the correct answer:

- Your activated score will be: a) 3 b) 4 **c) 5** d) 0
- Your teammate activated score will be: a) 3 b) 4 c) 5 **d) 6**
- Your team score will be: a) 3, b) 4, **c) 5, d) 6**
- Your earnings will be: a) less than your teammate's earnings, b) larger than your teammate earnings, **c) equal to your teammate earnings.**
- YOUR ACTIVATED SCORE: **[5]**
- TEAMMATE'S ACTIVATED SCORE: **[6]**
- TEAMSCORE: **[6/5]**
- Your earnings: **[£1.90/£1.65]**

Additional questions:

- Will you learn how many questions your teammate activated? Yes/**No**
- Will you learn how many questions you or your teammate answered correctly? Yes/**No**
- Will you learn your final earnings? **Yes**/No

Part 2: Activation Decisions

How many questions do you think your teammate will activate? (If you guess it right we will add £0.20 to your earnings)

Please choose how many questions you want to activate.

Bear in mind that each activated question will cost you £0.10 as explained before, so please make sure you are certain of your choice before pressing submit. You will not be able to go back to change your decision.

Execution of Part 1's Raven Task

Questionnaire

This is the end of Part 2. Before proceeding to the payment stage please fill in the following questionnaire.

- Gender
- Age
- Nationality
- Have you ever taken the *IQ Test/Pattern* Task before? Yes/No
- What are your thoughts on the task you completed? [Open Ended]
- *What do you think Pattern Task measures?* [Open ended - Only in Non-Ego treatment]
- *How accurate do you think IQ task measures person's general intelligence level?* [Open ended - Only in Ego treatment]
- Please tell us what determined how many questions you chose to activate? [Open ended]
- Suppose your teammate is called X. Please choose the number of the picture below which best describes your relationship with your teammate X based on your experience in this study?

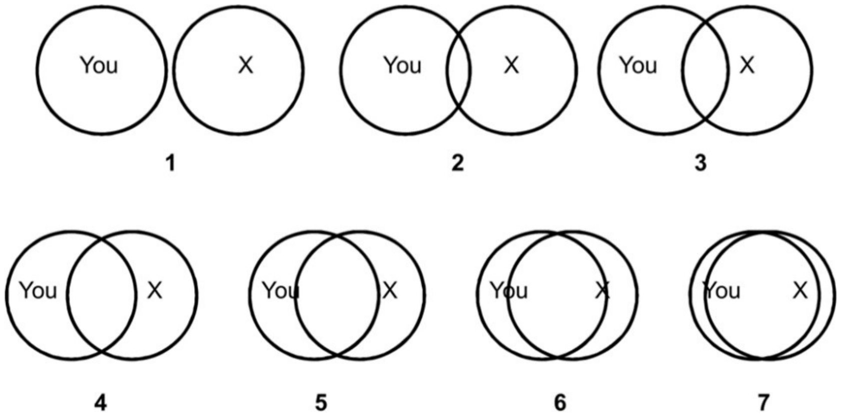

- Is there anything you would like us to know about your decision process during the experiment? [Open ended]

Would you like to learn what your score was in the *IQ/Pattern* Task? If yes, please choose which score you want to learn and we will show your scores in the final stage. This will cost you £0.10 each (to be subtracted from your earnings if you have positive earnings).

☐ Part 1: IQ/Pattern Task Score (costs £0.10)

☐ Part 2: IQ/Pattern Task Score (costs £0.10)

☐ Part 1 and 2: IQ/Pattern Task Score (costs £0.20)

☐ I do not want to learn my scores (costs £0.00)

Final Screen

Please provide your Prolific ID so that we can send to you results of your decisions and your final earnings amount.

You activated [ ] questions so your cost was [ ].

[If chosen to learn scores] – You answered [ ] and [ ] questions out of 10-items in your IQ/Pattern task. We will let you know of your TEAMSCORE and respectively your earnings once all participants have completed the study.
